# Hepatocellular Adenoma in a Patient with Ornithine Transcarbamylase Deficiency

**DOI:** 10.1155/2019/2313791

**Published:** 2019-10-02

**Authors:** Lin Cheng, Yajuan Liu, Wenjing Wang, J. L. Merritt, Matthew Yeh

**Affiliations:** ^1^Department of Pathology, Rush University Medical Center, Chicago, IL, USA; ^2^Department of Pathology, University of Washington, Seattle, WA, USA; ^3^Department of Pediatrics, University of Washington, Seattle, WA, USA; ^4^Department of Medicine, University of Washington, Seattle, WA, USA

## Abstract

Ornithine transcarbamylase (OTC) deficiency is an X-linked recessive disorder that leads to hyperammonemia and liver damage. Hepatocellular adenoma in OTC deficiency patients has not been previously described. Here we report the first such case to be described in the English language scientific literature.

## 1. Case Report

A 21-year-old woman with ornithine transcarbamylase (OTC) deficiency presented for liver transplant. She had no developmental abnormalities physically or intellectually. At age 13 years, she had acute behavioral changes (nonsensical word substitutions and combativeness) followed by episodes of nausea, vomiting, and abdominal pain. Initial laboratory evaluations revealed plasma ammonia above 200 *µ*M (reference range: 11–32 *μ*M), plasma glutamine 1542 *μ*M (reference range: 332–754 *μ*M), and trace levels of citrulline. Urine organic acid analysis showed elevated urine orotic acid. She was therefore diagnosed as a symptomatic heterozygous patient with OTC deficiency. Gene sequencing confirmed a duplication of exon 3 in *OTC* gene; testing in her mother was negative and her father had no history of suspicious symptoms of hyperammonemia. The patient was treated with dietary protein restriction, essential amino acid supplementation, oral sodium phenylbutyrate 100 mg/kg/day, and L-arginine powder 1000 mg three times per day. Later, she was switched to glycerol phenylbutyrate 5.5 mL three times per day; along with L-arginine 4000 mg three times per day. Due to refractory hyperammonemia on maximum therapy, oral sodium benzoate (10%) 100 mg/kg/day was added to help with ammonia control. Despite the increase of doses, her disease deteriorated with 3–6 hospitalizations per year with peak ammonia levels ranging from 100 to 350 *µ*M and plasma glutamine levels up to near 1200 *µ*M. Therefore, a living donor partial liver transplant was eventually performed.

**Figure 1:**
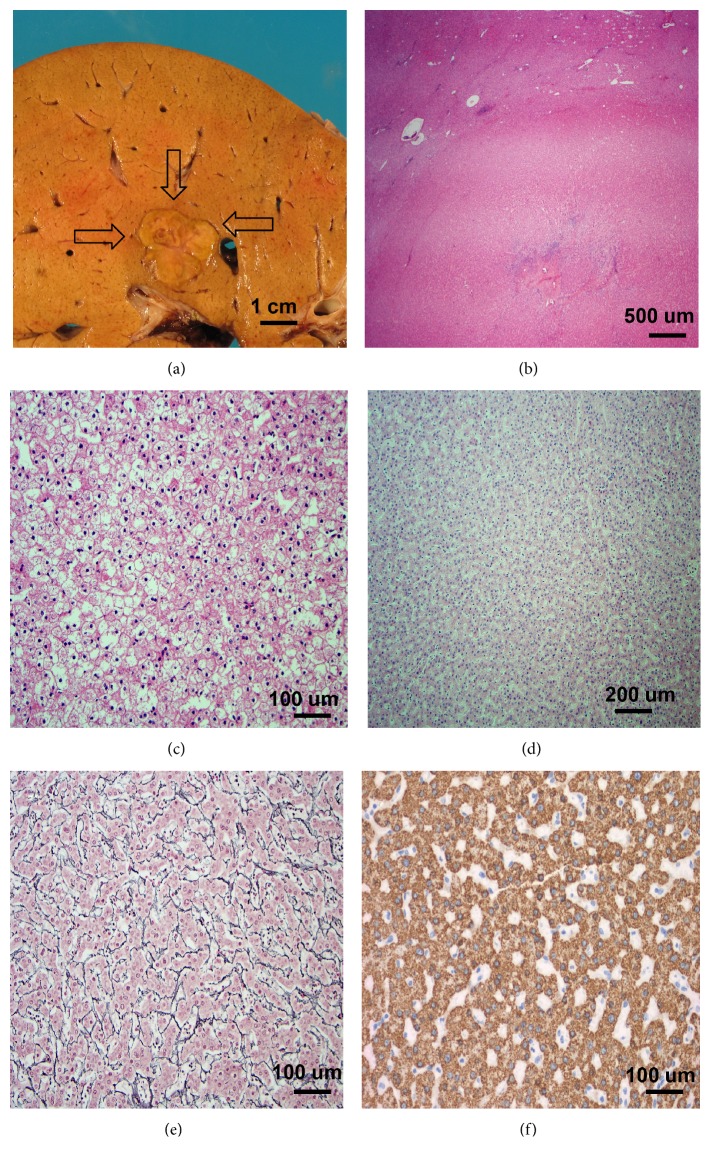
The explant liver from our patient with OTC deficiency contains a hepatocellular adenoma. (a) The tumor is a well-circumscribed, soft, tan nodule (shown by arrows), measuring 2.0 cm. (b) At low power magnification, the nodule is homogeneous without thick fibrous bands (H&E, 40x). (c) The background liver shows glycogen changes with clear to pink cytoplasm and distinct cell membrane (H&E, 200x). (d) The nodule is composed of hepatocytes with normal N/C ratios (H&E, 100X). (e) Reticulin stain highlights the normal reticulin framework and shows the hepatocellular plates of overall 1–2 cells in thickness (200x). (f) Immunostaining for HepPar1 shows diffuse immunoreactivity (200x).

The explanted liver weighed 1675 g and measured 18.0 × 25.0 × 8.5 cm, with attached gallbladder and a portion of falciform ligament. The outer surface of the liver was yellow-tan, smooth, and glistening. The gallbladder was filled with dark green viscous fluid and showed a smooth velvety mucosal surface with no calculi identified. The cut surface of the liver was yellow-brown, smooth, and homogeneous, with one nodule identified in segment 4/6. This nodule was well-circumscribed, soft, tan, and measured 2.0 × 2.0 × 1.5 cm. No other masses or lesions were identified (Figure 1(a)).

Histologically, in multiple areas of the liver parenchyma, hepatocytes were mildly enlarged and swollen, with clear and pale cytoplasm and distinct, enhanced cell borders, consistent with glycogen changes (Figure 1(b) and 1(c)). Within the nodule, there was no normal lobular architecture, portal tracts, or central veins present. The nodule was composed of clusters of hepatocytes forming cords of 1–2 cells thick, with normal nuclear to cytoplasmic ratio and bland-appearing nuclei (Figure 1(d)). There were no mitotic figures identified. Reticulin stain did not show abnormal hepatocellular network (Figure 1(e)). The lesional cells were immunoreactive to HepPar1, confirming hepatocytic origin (Figure 1(f)). A Masson trichrome stain showed no significant fibrosis. An iron stain was negative for abnormal iron deposition.

The above findings were suggestive of a hepatocellular adenoma (HCA). Differential diagnoses such as focal nodular hyperplasia (FNH) and hepatocellular carcinoma (HCC) had to be ruled out.

**Figure 2:**
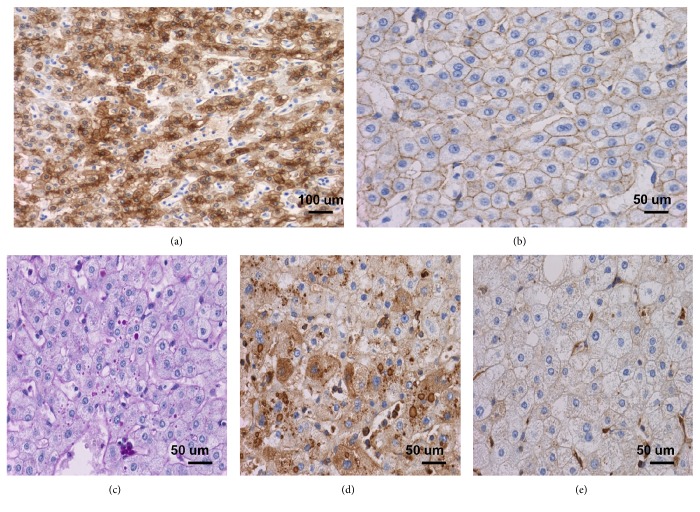
The hepatocellular adenoma is further subtyped as *β*-catenin activated HCA. (A) Immunostaining for glutamine synthetase shows diffuse reactivity within the tumor (200x), (B) *β*-catenin immunostain is negative for nuclear staining (400x). Intracytoplasmic globules in focal areas of the hepatocellular adenoma are observed. (C) PAS-D stain highlights small PAS positive and diastase resistant cytoplasmic globules (400x). These globules are immunoreactive with alpha-1-antitrypsin (D) but not alpha-1-antichymotrypsin (E) (400x).

There were rare fibrous septa with ductular reaction at the periphery of the nodule. No central fibrous scar or septa was seen. A glutamine synthetase immunostain was performed. The nodule lacked the classic map-like staining pattern typically seen in FNH [1]. Instead, it showed a diffuse heterogeneous cytoplasmic staining pattern (Figure 2(a)) that is commonly seen in *β*-catenin activated subtype HCA and HCC. Interestingly, *β*-catenin immunostain was negative for aberrant nuclear staining (Figure 2(b)). The hepatocytes within the nodule were also negative for both serum amyloid A (SAA) and C-reactive protein (CRP) immunostains. In focal areas within the nodule, Periodic acid Schiff (PAS) positive and diastase resistant cytoplasmic globules were observed. These globules were immunoreactive to alpha-1-antitrypsin but not alpha-1-antichymotrypsin (Figures 2(c)–2(e)), confirming that they are alpha-1-antitrypsin globules. They are not present in the non-tumor background liver. HCC commonly shows recurrent copy number aberrations and loss of heterozygosity (LOH) [2]. Cytogenomic microarray analysis (CMA) was performed by using the Illumina Infinium CytoSNP-850K BeadChip v1.1 (Illumina Inc., CA) with genomic DNA extracted from macrodissected formalin-fixed and paraffin-embedded tumor tissue. Allele and intensity ratio data of the fluorescent signals were generated and microarray data were visualized and analyzed using Nexus 8.0 (Biodiscovery Inc., CA) to identify chromosomal copy number alterations (CNAs) and regions of copy number neutral absence or loss of heterozygosity (cnLOH). CMA study showed that neither clonal CNAs nor copy neutral LOH were detected. This result was more consistent with the diagnosis of HCA and argues against HCC.

Therefore, our final diagnosis was a benign hepatocellular neoplasm, consistent with hepatocellular adenoma, *β*-catenin activated subtype (bHCA).

## 2. Discussion

The urea cycle primarily happens in the liver, which converts ammonia into a less toxic product—urea. The urea cycle was first discovered in 1932 by Hans Krebs and Kurt Henseleit [3]. It includes two mitochondrial and three cytosolic reactions. Each reaction is catalyzed by a specific enzyme, and deficiency of any of these enzymes can cause urea cycle disorders [4]. Ornithine transcarbamylase (OTC) is located within mitochondria. It converts carbamoyl phosphate and ornithine into citrulline [4]. OTC deficiency is the most common urea cycle disorders in humans, with an incidence of less than 1/80,000. It is inherited in an X-linked recessive pattern; therefore, most patients are male. Female heterozygotes usually are asymptomatic; however, approximately 20% of female carriers are symptomatic. The exact mechanism is not clear, but skewed/unfavorable X-inactivation that silences the normal X chromosome may be one of the reasons [5].

Diagnosis of OTC deficiency is based on clinical suspicions and biochemical testing. Molecular testing can confirm the diagnosis in about 90% of OTC deficiency patients. To date, 417 pathogenic variants have been identified in *OTC* gene [6]. These pathogenic variants cause variable severity of clinical presentations, ranging from early-onset severe hyperammonemia in newborns to late-onset intermittent hyperammonemia in adults. Currently, the treatment of OTC deficiency is focused on avoiding hyperammonemic episodes. The management includes low-protein diet and nitrogen-scavenging drugs for long-term ammonia level control, and dialysis and hemofiltration in acute attack. Initial gene therapy delivered by adenoviral vector was attempted in 1990s but was stopped due to an unexpected death [7]. A new gene therapy phase 1/2 clinical trial in adults with late-onset OTC deficiency was launched in 2017, which uses a self-complementary adeno-associated viral vector (scAAV). This trial will follow patients for 5 years in total to evaluate the long-term safety and efficacy of the therapy [8]. For patients who have refractory hyperammonemia, liver transplant prevents future hyperammonemia.

In OTC-deficient patients, liver damage is common, such as inflammation, glycogen changes, steatosis, and cholestasis [9]. Acute liver failure has been reported as well [10, 11]. However, liver tumors, such as HCA or HCC, are very rare. There was one case of HCC that occurred in an OTC-deficient patient, 14 years after she received gene therapy with adenoviral vector [12]. Although the wild-type adenovirus is considered oncogenic, the genetically engineered adenoviral vector is replication defective and is not considered high risk for HCC [12]. In addition, no vector genome was found in tumor or non-tumor tissues in this patient's liver. Therefore, the hepatocellular carcinoma was likely caused by the metabolic complications of OTC deficiency rather than the adenoviral vector [13]. The ongoing clinical trial uses an adeno-associated virus vector that can insert its DNA into human genome, but it is still not established whether adeno-associated virus genome insertion is tumorigenic [14, 15]. HCA in OTC-deficient patients has not been previously reported.

Hepatocellular adenoma (HCA) is a benign neoplasm of liver, with an incidence of 3/1,000,000 per year in Europe and North America [16]. The overall frequency of malignant transformation from HCA to HCC is about 4.2% [17]. HCA occurs commonly in patients with the usage of anabolic steroids or androgenic steroids, women taking oral contraceptive pills (OCP), type I diabetes, beta-thalassemia, and glycogen storage diseases type 1 and 3. HCA can be single or multiple; multiple HCAs are commonly seen in patients with glycogen storage diseases; and adenomatosis is defined when there are more than 10 HCAs within the liver. While our patient has taken OCP for 5 years (which is a risk factor), it is not convincing that OCP alone would predispose her to the development of HCA. OCP is commonly used in female patients with OTC deficiency to prevent the decompensation caused by menstruation [18], but no HCA cases have been reported in such patient group.

The subclassification of HCA is based on the molecular and immunohistochemical features. Initially, 4 subtypes of HCAs were identified [19]: *HNF1A* mutated HCA (HHCA), *β*-catenin activated HCA (bHCA), inflammatory HCA (IHCA), and unclassified HCAs (UHCA). In 2017, Nault et al. proposed to further divide HCAs into 8 subtypes [20]. Different subtypes of HCAs can coexist in the same patient [21].

The HHCA subtype can be easily recognized on histology by the presence of moderate to severe steatosis, usually sparing the arterialized zones. Molecular studies show that HHCA contains pathogenic variants in *HNF1A* gene. HNF1A upregulates the expression of liver fatty acid binding protein (L-FABP) in normal livers. So, the loss-of-function *HNF1A* mutation leads to loss of L-FABP protein expression and negative L-FABP immunostain [22]. Most HHCAs are negative for inflammatory proteins (SAA and CRP) and glutamine synthetase [22].

The bHCA subtype has the highest risk of malignant transformation to HCC. Occasionally, pseudoacinar formations and cytological atypia are seen in bHCA, making it difficult to differentiate from HCC. The molecular feature of bHCA is the activation of the WNT/*β*-catenin pathway, which occurs mainly through pathogenic variants in *β*-catenin encoding gene *CTNNB1*. The pathogenic variants prevent the degradation of *β*-catenin protein, resulting in upregulation of its target genes such as *GLUL* (encoding glutamine synthetase). Therefore, immunohistochemically aberrant *β*-catenin nuclear staining and diffuse positive glutamine synthetase staining are indicators of WNT/*β*-catenin pathway activation. In addition, the glutamine synthetase diffuse staining pattern can be observed when genetic alterations occur in other members of the pathway without *CTNNB1* pathogenic variants, such as *RSPO2* gene rearrangement [23]. Therefore, immunostaining for glutamine synthetase is a better surrogate marker than immunostaining for *β*-catenin to predict activation of the WNT/*β*-catenin pathway.

The IHCA subtype usually shows sinusoidal dilatation, pseudoportal tracts containing thick-walled arteries, ductular reactions, and inflammatory cell infiltration on histological examination. Molecular study shows JAK/STAT pathway activation, such as gain-of-function pathogenic variants of IL-6 signal transducer gene (*IL6ST*). Immunohistochemically, IHCA shows strong positivity for SAA and CRP.

The UHCA subtype includes all the HCAs lacking the aforementioned specific morphologic and molecular features, and is supposed to be a diagnosis of exclusion. Interestingly, Henriet et al. showed overexpression of argininosuccinate synthase isoform 1 (ASS1) in all 17 UHCA cases by proteomic analysis [24]. Therefore, ASS1 immunostain could be considered as a marker for UHCA, if larger studies confirm the universal overexpression of ASS1 in UHCAs.

In 2017, Nault et al. [20] further divided bHCAs based on molecular study. Pathogenic variants in *CTNNB1 *exon 3 showed increased risk of malignant transformation (10%), and these HCAs were grouped as b^ex3^HCA. Pathogenic variants in *CTNNB1* exon 7 and 8 only mildly activated the WNT/*β*-catenin pathway with no increased risk for HCC, so they were grouped as b^ex7,8^HCA. In addition, studies showed that about half of the bHCAs had additional features of IHCA with JAK/STAT pathway activation, therefore these lesions with mixed features were called b-IHCAs, and they were further classified as b^ex3^IHCA and b^ex7,8^IHCA according to the *CTNNB1* pathogenic variants. For the UHCAs showing sonic-hedgehog pathway activation and association with obesity and increased risk of tumor hemorrhage, the lesions were reclassified as shHCA [20]. In summary, Nault et al. suggested using L-FABP, SAA, and glutamine synthetase immunostains for the initial subtyping of HCAs, and then employing molecular studies on WNT/*β*-catenin pathway, JAK/STAT pathway, and sonic- hedgehog pathway to further subtype HCAs.

In our case, molecular studies were negative for HNF1a or JAK/STAT3 mutations, so HHCA and IHCA subtypes were excluded. The *β*-catenin immunostain was negative for aberrant nuclear staining, but the glutamine synthetase immunostain showed diffuse heterogeneous positivity. Due to the limited amount of tissue available for molecular study after the analysis of negative HNF-1 and JAK/STAT-3 mutation, no remaining tissue was available to evaluate the *CTNNB1* pathogenic variants, but given the diffuse glutamine synthetase staining pattern, our case is best classified as bHCA.

OTC deficiency is not known as a direct cause for HCA. The possible mechanisms of HCA pathogenesis include chronic liver damage by toxic metabolites [25] and metabolism shifting such as abnormal glycogen accumulation [26]. The abnormal glycogen accumulation may be caused by the carbohydrate rich diet and amino acid supplementary. Further investigation is needed to better understand the tumorigenesis in OTC-deficient patients. Although there is no established protocol, surveillance in OCT-deficient patients for liver mass by interval ultrasound is recommended, especially in patients with longstanding disease.

The significance of alpha-1-antitrypsin globules within the HCA (which had not been previously reported) in this case is not certain. Our patients serum alpha-1-antitrypsin had never been checked as she never had persistent elevation of AST, ALT and bilirubin. While the intracytoplasmic globules are characteristic in patients with alpha-1-antitrypsin deficiency, they are not specific and are also commonly observed in cirrhotic liver [27] or hepatocellular carcinoma irrespective of etiology [28]. This may be attributed to impaired protein secretion in diseased liver. Alternatively, either monoallelic pathogenic variants or heterozygous biallelic variants of alpha-1 antitrypsin is possible in this patient, but the absence of alpha-1 antitrypsin globules in the non-tumor liver argues against this possibility.

## 3. Conclusion

OTC deficiency is the most common urea cycle disorder in humans. It causes hyperammonemia in patients and leads to multiple system damages including neural system and liver. Solid masses such as HCCs are rare in OTC-deficient patients. Here, we report the first case of OTC-deficient patient with an incidental HCA. Possible etiologies of her HCA include chronic liver damage by toxic metabolites and abnormal glycogen accumulation. Additional studies of similar cases would help us further understand the mechanism of HCA pathogenesis in OTC-deficiency patients, improve the current surveillance strategy and treatment regime, and prevent the generation of HCAs and HCCs in their livers.
